# Sleep and Nutrition Interactions: Implications for Athletes

**DOI:** 10.3390/nu11040822

**Published:** 2019-04-11

**Authors:** Rónán Doherty, Sharon Madigan, Giles Warrington, Jason Ellis

**Affiliations:** 1 Letterkenny Institute of Technology, Port Road, Letterkenny, F92 FC93 County Donegal, Ireland; 2 Sport Ireland Institute, National Sport Campus, Abbotstown, 15, D15 Y52H Dublin, Ireland; smadigan@instituteofsport.ie; 3 Northumbria Centre for Sleep Research, Northumbria University, Newcastle NE1 8ST, UK; jason.ellis@northumbria.ac.uk; 4 Health Research Institute, Schuman Building, University of Limerick, V94 T9PX County Donegal, Ireland; giles.warrington@ul.ie; 5 Department of Physical Education and Sport Sciences, University of Limerick, V94 T9PX County Donegal, Ireland

**Keywords:** sleep, athletes, chrononutrition

## Abstract

This narrative review explores the relationship between sleep and nutrition. Various nutritional interventions have been shown to improve sleep including high carbohydrate, high glycaemic index evening meals, melatonin, tryptophan rich protein, tart cherry juice, kiwifruit and micronutrients. Sleep disturbances and short sleep duration are behavioural risk factors for inflammation, associated with increased risk of illness and disease, which can be modified to promote sleep health. For sleep to have a restorative effect on the body, it must be of adequate duration and quality; particularly for athletes whose physical and mental recovery needs may be greater due to the high physiological and psychological demands placed on them during training and competition. Sleep has been shown to have a restorative effect on the immune system, the endocrine system, facilitate the recovery of the nervous system and metabolic cost of the waking state and has an integral role in learning, memory and synaptic plasticity, all of which can impact both athletic recovery and performance. Functional food-based interventions designed to enhance sleep quality and quantity or promote general health, sleep health, training adaptations and/or recovery warrant further investigation.

## 1. What is Sleep?

Sleep, in humans, is defined as a complex reversible behavioural state where an individual is perceptually disengaged from and unresponsive to their environment [1]. Sleep architecture has two basic states based on physiological parameters: non-rapid eye movement sleep (NREM) and rapid eye movement (REM) sleep [2]. Sleep stages fall along a continuum from fully awake to deep sleep [3]. NREM has been defined as “a relatively inactive yet actively regulating brain in a moveable body” [2], (p.17). In terms of brain activity, the electroencephalogram (EEG) pattern of NREM sleep is commonly described as synchronous (increasing depth of sleep is indicated by progressive dominance of high voltage, low frequency EEG patterns), with characteristic waveforms (sleep spindles, K-complexes and high voltage waves) [2]. NREM is usually associated with minimal or fragmented mental activity. Table 1 shows the traditional four stages of NREM which are associated with differing levels of depth of sleep, with arousal thresholds generally lowest in Stage 1 and highest in Stage 4 sleep [2].

In contrast, REM sleep is defined by EEG activation, muscle atonia (paralysis) and episodic bursts of rapid eye movement [2]. REM sleep is associated with cognitive activity, while brain stem mechanisms inhibit spinal motor neurons limiting movement. Hence, REM sleep has been defined as “an activated brain in a paralysed body” [2], (p.16). It should be noted that the American Academy of Sleep Medicine (AASM) have recommended alternative terminology for Sleep staging. Wake is referred to as W, NREM sleep is referred to as N and is divided into three stages: N1 – Stage 1, N2 – Stage 2 and N3 – Slow Wave Sleep or Deep Sleep, i.e., Stage 3 and 4 combined; while REM is referred to as R [5]. 

Sleep health is a multidimensional pattern of sleep-wakefulness adapted to individual, social and environmental demands, which promotes physical and mental wellbeing [6]. Good sleep health is characterised by satisfaction, appropriate timing, adequate duration, high efficiency and sustained alertness during waking hours [6]. Sleep deprivation adversely affects glucose metabolism and neuroendocrine function which can affect carbohydrate metabolism, appetite, energy intake and protein synthesis [1]. These factors may negatively impact an athlete’s nutritional, metabolic and endocrine status impacting athletic performance and recovery [1], (e.g., impaired glucose metabolism could reduce glycogen repletion while impaired protein synthesis could reduce recovery and adaptation from training). This narrative review examines and evaluates the interaction between nutrition and sleep.

### How and Why Sleep Occurs

The brain is essentially an electrical system with circuits that switch on and off to promote either wakefulness or sleep. Since the arousal and sleep-promoting systems are mutually inhibitory, a sleep switch or ‘flip-flop’ model has been proposed [7]. A flip-flop switch contains mutually inhibitory elements where activity in one of the competing sides shuts down inhibitory inputs from the other side producing two discrete states with sharp transitions [8]. Activation of arousal systems inhibits sleep active neurons facilitating sleep while activation of sleep-promoting neurons inhibits arousal-related neurons reinforcing consolidated sleep episodes providing a mechanism for stabilisation of sleep and waking states [9].

The circadian rhythm in humans has been estimated in young males (24.18 ± 0.04 h; PCV 0.54%) and older adults (24.18 ± 0.04 h; PCV 0.58%), low percentage coefficients of variation and no significant difference between the groups indicated a small range variability in circadian rhythms [10]. Humans however, typically display individual differences in their behaviour (e.g., social activities, daytime activities and sleep). Chronotype is the expression of individual circadian rhythmicity and has been categorised as follows: morning types, intermediate types and evening types [11]. Chronotype is, in part, genetic but cultural and environmental factors also affect an individual’s sleep pattern. Research in the general population has demonstrated that most people are intermediate types (70%) with the remainder being either morning types (14%) or evening types (16%) [12]. 

Sleep is a dynamic process largely regulated by two factors; the circadian systems and the sleep homeostat. The Two Process Model for Sleep Regulation was developed to illustrate the interaction of the homeostatic sleep drive (sleep pressure or urge to sleep that accumulates during wakefulness) and the circadian system (endogenous timing system) in the timing and duration of sleep [13,14]. The homeostatic process (S) is a function of sleep and waking, while the circadian process (C) is controlled by a circadian oscillator [10]. S increases during waking and declines during sleep and it interacts with C, which is independent of sleep and waking and receives cues (e.g., light) from the environment [13,14]. The suprachiasmatic nucleus (SCN) in the brain is central to this process but secondary clock systems have been identified throughout the body [14]. 

Process S is an endogenous mechanism, relying on exogenous cues to regulate it to approximately 24 h. Process S represents sleep debt which increases during waking and reduces during sleep within a range that oscillates within a period that is normally entrained to day and night by process C [14]. When S reaches the lower boundary of the range, awakening is triggered and when S reaches the upper boundary sleep is triggered [14]. In terms of process C, the Two-Process Model focuses on time-of-day effects on sleep propensity, specifically that sleep propensity is minimal near midday and is strongly promoted in the early hours of the morning [13]. This circadian rhythmicity in sleep propensity is combined with S by C dictating the threshold values at which S transitions from sleep to wake, and vice versa [13,14]. Core body temperature and melatonin rhythms are markers of C [11]. The SCN has melatonin receptor cells, as darkness falls, melatonin is secreted by the pineal gland making the individual sleepy [15]. Animal studies have demonstrated that exogenous melatonin and ramelteon (an MT1/MT2 melatonin receptor agonist) function as non-photic entrainers, which phase advance the SCN [16]. A Three-Process Model of Sleep Regulation has also been proposed whereby sleepiness and alertness are stimulated by the combined action of a homeostatic process, a circadian process and sleep inertia process, the model has been extended to include sleep onset latency (the length of time of the transition from wakefulness to sleep), sleep length and performance [17]. 

Sleep has a restorative effect on the immune system and the endocrine system, facilitates the recovery of the nervous and metabolic cost of the waking state and has an integral role in learning, memory and synaptic plasticity (ability of synapses to strengthen or weaken over time) [18,19]. Sleep, particularly slow wave sleep (or N3) early in the night promotes prolactin release, while the anti-inflammatory actions of cortisol and catecholamines are reduced [18]. Acute sleep deprivation and sleep disturbance (short sleep duration or reduced sleep efficiency) impair adaptive immunity which is associated with reduced response to vaccinations and increased vulnerability to infectious diseases, attributed to reduced growth hormone release during deep sleep and increased sympathetic output [20]. Tumour necrosis factor-α (TNFα) along with other cytokines are considered key to the regulation of sleep in normal physiological conditions [21]. Research has demonstrated that sleep disturbance (i.e. insomnia) and extremes of sleep durations affect risk factors of inflammatory disease and contribute to all-cause mortality [18,22]. Increased levels of circulating inflammatory markers (i.e., C-reactive protein [CRP] and Interleukin-6 [IL-6]) predict body mass gain in older adults [23] and type 2 diabetes [24]. Sleep disturbance is believed to have proximal effects on IL-6, which induces CRP [18], therefore, increases in CRP may be attributed to persistent or severe sleep disturbance. In a recent meta-analysis sleep disturbance (i.e., poor sleep quality, insomnia) was associated with increased levels of IL-6 (ES: 0.20 (0.08–0.31)) and CRP (ES: 0.12 (0.05–0.19)) [18]. Short sleep duration (< 7 h per night) was associated with increased IL-6 (ES: 0.29 (0.05–0.52)), while long sleep duration (> 8 h per night) was also associated with increased IL-6 (ES: 0.11 (0.02–0.20)) but also increased CRP (ES: 0.17 (0.01–0.34)) [18]. Similarly, a meta-analysis of sleep duration and all-cause mortality demonstrated a U-shaped association, whereby long sleep (> 8 h per night) has a 30% (RR: 1.30 (1.22–1.38)) greater risk while short sleep (<7 h per night) has a 12% (RR: 1.12 (1.06–1.18)) greater risk compared to normal sleep reference (7–8 h per night) [25].

Inappropriate timing of lifestyle behaviours can cause disruption to the circadian rhythm, resulting in an altered physiological response (e.g., poor sleep). Lifestyle factors (e.g., caffeine consumption, alcohol consumption and timing of sleep) can cause alterations in environmental cues which may negatively impact circadian rhythms and in turn result in negative physiological consequences [26]. The SCN receives environmental cues such as the light-dark cycle and additional information from other areas of the brain (e.g., when we eat or exercise). Give that Process C can be modified by exogenous cues [27], there is scope for investigation of nutrition interventions to enhance sleep quality and quantity. Similarly, the effect of nutrition interventions that promote athlete recovery on sleep quality and quantity should be investigated. 

## 2. Sleep and Athletes

The classic view of sleep is that it is a recovery process, with the circadian system regulating feelings of sleepiness and wakefulness throughout the day [28]. Cognition, tissue repair and metabolism are critical psychological and physiological factors that contribute to training capacity, recovery and ultimately performance [28]. The relationship between sleep, performance and recovery can be viewed in terms of 3 key factors that affect the recuperative outcome:

1. Sleep length (total sleep duration; hours/night, plus naps)
2. Sleep quality (i.e., the experience and perceived adequacy of sleep)
3. Sleep phase (circadian timing of sleep) [28].

Post-exercise recovery is vital for all athletes. If the balance between training stress and physical recovery is inadequate, performance in subsequent training sessions or competition may be adversely affected [15]. Muscle fatigue or soreness may adversely affect sleep, with inflammatory cytokines linked to disruption of normal sleep [29]. Inadequate recovery can reduce autonomic nervous system (ANS) resources, with an associated reduction in heart rate variability (HRV) and increased resting heart rate [30]. Sleep deprivation is associated with increased catabolic and reduced anabolic hormones which results in impaired muscle protein synthesis [31], blunting training adaptations and recovery. 

Sleep disturbances and inadequate sleep duration have been reported in athletic populations. Assessment of the sleep patterns of professional male ice hockey players (*n* = 23) using polysomnography (PSG), demonstrated mean total sleep duration was 6.92 h; 95% CI 6.3–7.5 h [32]. Similarly, sleep was self-reported as the most important recovery modality utilised by South African athletes (*n* = 890; international *n* = 183, national *n* = 474, club *n* = 233) [15]. While a similar study found that 66% (*n* = 416) of elite German athletes (*n* = 632) reported pre-competition insomnia symptomology including difficulty falling asleep, waking during the night and early final waking times [33]. Sleep duration (< 8 h) has been identified as the strongest predictor of injury in adolescent athletes (RR = 2.1; 95% CI: 1.2–3.9) [34]. The Karolinska Athlete Screening Injury Prevention (KASIP) study investigated injury occurrence in Swedish adolescent elite athletes (*n* = 340; 178 males and 162 females) and demonstrated that athletes sleeping >8 h were less likely to suffer an injury (OR: 0.39; 95% CI 0.17–0.96) [35]. The aetiology of sleep disturbances is unclear during periods of intense training, it is unclear whether poor sleep is a symptom of overtraining, or intense training negatively affects sleep and recovery [30]. Sleep also has a pivotal role to play in performance, training adaptations and recovery [1,18]. Given the importance of sleep for athlete recovery, further research is warranted to investigate potential nutritional interventions to promote improved sleep quality and/or duration and recovery in athletes. 

### 2.1. Sleep, Nutrition and Athletes

Nutrition support needs to be periodised in relation to the demands of the athlete’s daily training and overall nutritional goals [36]. The focus of ‘training’ nutrition is to promote adaptations while the focus of ‘competition’ nutrition is optimal performance [36]. Athletes also have added responsibility to adhere to the World Anti-Doping Agency (WADA) code and are subject to testing for prohibited substances. If an athlete chooses to take any supplement they must do so in a safe and effective manner. Athletes should check that any supplement they take has been tested for banned substances, and independent testing programmes (e.g., Informed Sport and Informed Choice) offer additional protection. Athletes are advised to seek the professional advice of a qualified sports dietician/nutritionist regarding any nutritional supplement. Training adaptations and recovery can be maximised by optimal nutrition practices or impaired by suboptimal nutrition practices [36,37,38]. Nutrients such as carbohydrate (high glycaemic index evening meal reduced sleep onset latency), protein (consumption of dairy sources may increase sleep duration), ethanol (reduced REM sleep) [37] and caffeine (increased sleep onset latency, reduced total sleep duration and reduced sleep quality) [39], as well as the timing and quantity of meals (large portions and/or meals later in the evening can negatively impact sleep potentially due to the thermogenic effect of digestion) can affect circadian rhythms [40]. Caffeine consumption can lead to poor sleep which, in turn, can lead to increased caffeine consumption. Caffeine increases the state of alertness, antagonising adenosine receptors, which also leads to a reduction in the inclination to sleep [39]. Alcohol consumption has been associated with poorer sleep quality and quantity, reduced REM sleep and increased sleep disturbance in the second half of the sleep bout [41]. Similar to nutrition, sleep disturbances (difficulty initiating or maintain sleep) and sleep deprivation (not getting enough sleep) are risk factors for inflammation [18,42], which can be treated or managed to promote recovery and/or performance. For sleep to have a restorative effect on the body, it must be of adequate duration which is dependent on age [28,42]. Sleep recommendations particularly the amount of sleep required, change over the lifespan from adolescents (8–10 h), adults (7–9 h), and older adults (7–8 h) [42].

### 2.2. Chrononutrition

Recently the term Chrononutrition has been used to describe the interaction between food and the circadian system [39]. It has been suggested that the internal clock can be altered by changing the timing and nature of food intake [39]. Chrononutrition has been characterised as including two aspects:

1. Timing of food intake or contributions of food components to the maintenance of health; and
2. Timing of food intake or contributions of food components to rapid changes in or resetting of a human’s system of internal clocks [39].

Several neurotransmitters are involved with the sleep-wake cycle including 5–hydroxytryptophan (5-HT), GABA, orexin, melanin concentrating hormone, cholinergic, galanin, noradrenaline and histamine [7]. Therefore, nutrition interventions that act on these neurotransmitters could positively impact sleep. Dietary precursors can influence the rate of synthesis and function of neurotransmitters (e.g., serotonin synthesis is dependent on the availability of its precursor tryptophan in the brain) [1]. Tryptophan is transported across the blood brain barrier by a system that shares transporters with several large neutral amino acids (LNAA) [1]. The ratio of tryptophan:LNAA in the blood is vital to the transport of tryptophan into the brain and can be increased through consumption of tryptophan, a high carbohydrate/low protein diet or α-lactalbumin (whey derived protein) [43].

### 2.3. Carbohydrate

Carbohydrate consumption has been shown to increase plasma tryptophan concentrations [44]. Carbohydrates affect plasma tryptophan:LNAA ratio and may compliment the sleep enhancing effect of consuming tryptophan rich protein [40]. Insulin influences the transport of tryptophan across the blood brain barrier after a carbohydrate rich meal, as it is an anabolic agent it also facilitates the uptake of LNAA by muscle [26]. Consumption of high glycaemic index (GI) carbohydrate increases the ratio of circulating tryptophann:LNAA via direct action of insulin which promotes muscle uptake of LNAA [45]. This increases tryptophan availability for synthesis of serotonin and ultimately melatonin. GI has been shown to affect sleep onset latency (length of time of the transition from wake to sleep) [44]. A high GI meal consumed four hours before bed, significantly (*p* = 0.009) reduced sleep onset latency (9.0 ± 6.2 min) compared to a low GI meal (17.5 ± 6.2 min) and the same meal consumed 1 hour before bed (14.6 ± 9.9 min) [44]. Among a large sample (*n* = 4452) from the National Health and Nutrition Examination survey lower carbohydrate intake (24–h recall and structured interview) has been significantly associated (OR 0.71; 0.55–0.92, *p* = 0.01) with insomnia symptoms (difficulty maintaining sleep) [46]. Consumption of a high-carbohydrate meal (130 g) when compared to a low-carbohydrate meal (47 g), or a meal containing no carbohydrate, 45 min before bedtime increased REM and decreased light sleep and wakefulness [47]. The timing of carbohydrate evening meals and the carbohydrate content of evening meal on sleep and athlete recovery requires further investigation within athletic populations. 

### 2.4. Melatonin

Melatonin is a hormone secreted by the pineal gland, that has displayed sedative effects [48,49]. Since endogenous melatonin influences core temperature facilitating sleep, increased exogenous melatonin could affect changes in core temperature improving sleep quality [50]. However, the effect is relative to the person’s endogenous melatonin levels. In many Western countries, Cow’s milk has traditionally been considered a sleep promoting beverage. Melatonin is a naturally-occurring compound in cow’s milk, but its concentration increases significantly if cows are milked in darkness at night referred to as ‘night time milk’ [40]. Increased tryptophan and melatonin concentrations appear to be the property responsible for the sleep promoting effect of night time milk. Consumption of night time milk (melatonin concentration of 39.43 pg/mL) compared to daytime milk (4.03 pg/mL) significantly increased circulating melatonin (26.5%) concentration in rats [51], indicating that high melatonin concentrations are necessary for milk to affect blood melatonin concentrations. When the night time milk was supplemented with tryptophan (2.5 g/L) circulating melatonin concentrations significantly increased further (35.5%) [51]. 

Melatonin has extremely low toxicity even at relatively high doses and can easily cross physiological barriers due to its optimal size, partial water solubility and high lipid solubility however, it must be noted that there appears to be no added benefit to doses >3 mg [49]. Ingestion of melatonin affects sleep propensity and has hypnotic effects enhancing sleep quality and duration, pharmacological melatonin can be used to manipulate circadian timing [49]. A positive effect of low doses (0.3 mg or 1 mg) of exogenous melatonin (gelatin capsules) on sleep onset latency has been observed in a small group of healthy males (*n* = 6), when administered at either 6:00 pm (0.3 mg 16.5 ± 19.9 min; 1 mg 12.3 ± 13.6 min; Placebo 23.1 ± 22.7 min) and 8:00 pm (0.3 mg 19.6 ± 14.1 min; 1 mg 20.7 ± 17.7 min; Placebo 53.4 ± 51.9 min) [52]. However, the impact was time dependent as a 0.3 mg dose increased sleep onset latency when consumed at 9:00 pm (0.3 mg 25.1 ± 10.5 min; 1 mg 12.1 ± 7.4 min; Placebo 8.8 ± 4 min) and there was no evidence of an effect when the 1 mg dose was administered at 9:00 pm [52]. The results indicate a low dose of melatonin similar to nocturnal physiological concentrations can elicit a sleep-inducing effect. A dose response relationship was not evident as the 0.3 mg dose, which is similar to endogenous melatonin concentrations, was as effective as the 1 mg dose when administered at 6:00 pm or 8:00 pm. 

### 2.5. Tryptophan Rich Protein

Tryptophan is an essential amino acid that is a precursor to serotonin and melatonin, which can cross the blood-brain barrier by competing for transport with other LNAA [1]. Conversion to serotonin is dependent on sufficient precursor availability in the brain, an increase in brain tryptophan occurs when the ratio of free tryptophan to branched chain amino acids increases, following tryptophan conversion to serotonin, melatonin is produced [1]. Dietary sources of tryptophan include milk, turkey, chicken, fish, eggs, pumpkin seeds, beans, peanuts, cheese, and leafy green vegetables. Dietary tryptophan has been shown to improve sleep, in a comparison of food bars (Food 1: 25 g deoiled butternut squash seed meal and 25 g dextrose, Food 2: 250 mg of pharmaceutical tryptophan and Food 3: 50 g rolled oats [control]) [53]. Food 1 and Food 2 produced significant results (*p* ≤ 0.05) for reduction of time awake during the night (19.2% and 22.1%), increased sleep efficiency (% of time spent in bed; asleep) (5.19% and 7.36%) and increased subjective sleep quality (12.2% and 11.8%) [53], indicating that relatively small doses (250 mg) of dietary tryptophan can positively impact sleep. The milk protein, α-lactalbumin has been reported as having the highest natural levels of tryptophan among all protein food sources [54]. Ingestion of α-lactalbumin enriched whey protein, significantly (*p* < 0.05) increased tryptophan:LNAA by 48% compared to a casein enriched diet [54]. In a similar study, healthy adults (*n* = 14) with sleep complaints consumed milkshakes containing either α-lactalbumin (20 g) or a casein placebo. Evening ingestion of α-lactalbumin resulted in a 130% increase in tryptophan:LNAA prior to bed and modest but significant reduction in morning sleepiness and improved alertness the following morning [55]. Tryptophan depletion studies have demonstrated decreased tryptophan plasma concentrations affected sleep fragmentation (arousal index (events/h)), REM sleep latency (the interval between first epoch of stage 2 and the first epoch of REM sleep), and REM density (the cumulated duration of each REM burst divided by the duration of each REM sleep period) compared to baseline and placebo [56,57]. Consumption of tryptophan rich protein (e.g., milk) could affect changes in core temperature improving sleep quality [51]. The effects of tryptophan rich protein (e.g., α-lactalbumin enriched whey and casein) interventions on sleep and recovery, warrant further investigation.

### 2.6. Antioxidants

Both the general population and athletes can benefit from nutritional and supplementation support to boost immunity and reduce acute and chronic inflammation during periods of increased training load and competition. Antioxidants are any substance that significantly delay or prevent oxidative damage of a target molecule [58]. The fact that exercising muscles produce free radicals has motivated many athletes to consume antioxidant supplements in an attempt to reduce exercise induced free-radical damage and/or muscle fatigue. The antioxidant capacity of several dietary micronutrients is an emerging area of interest to support the endogenous antioxidant defence system of athletes and attenuate the negative effects of oxidative damage due to free radicals. Antioxidant consumption may influence recovery from exercise but may also influence sleep since sleep regulation is influenced by pro-inflammatory cytokines [59]. Dietary antioxidants (e.g., vitamin C and vitamin E) augment endogenous antioxidant content within skeletal muscle [59]. Vitamin E is a fat-soluble vitamin made up of several isoforms known as tocopherols, with α-tocopherol being the most active and abundant [60]. Vitamin E is an important antioxidant due to its abundance within cells, mitochondrial membranes and its ability to act directly on reactive oxygen species [60]. Vitamin E reacts with other antioxidants such as vitamin C, beta-carotene and lipoic acid, which have the capacity to regenerate vitamin E from its oxidised form. However, supplementation with high doses (800 IU/day) of vitamin E did not counteract OS in triathletes (*n* = 38), the intervention group demonstrated significantly (*p* ≤ 0.05) higher levels of post-race inflammation and OS (Plasma F_2_-isoprostanes increased 181% versus 97% and IL-6 166 ± 28 pg∙mL^–1^ versus 88 ± 13 pg∙mL^–1^) than the control group [60]. Interestingly, despite increased markers of OS and inflammation in the intervention group, there was no significant difference between the groups in terms of race performance. 

Vitamin A is a fat-soluble vitamin present in many lipid substances, beta-carotene can be converted into vitamin A, when necessary, from within the body [61]. Vitamin C is a water-soluble vitamin and is extremely effective in extracellular fluids, but is also effective in the cytosol [61]. It must be noted that antioxidants are heterogeneous, they function in a distinct manner and do not solely regulate ROS [61]. Consumption of an antioxidant does not guarantee that the compound will act as an antioxidant within the body, therefore positive findings from one antioxidant or combination of antioxidants cannot be generalised [59]. It has been suggested that a high intake of antioxidants could potentially reduce training adaptations. It is accepted that repeated exercise bouts (i.e. training) induce disruption in skeletal muscle homeostasis that regulate training adaptations [62,63]. While it has been reported that high doses of antioxidants could reduce training adaptations of muscle mitochondrial biogenesis and VO_2max_, not all antioxidant studies have demonstrated negative effects and it has been suggested that the specific antioxidant used, the dose and timing of ingestion all affect outcomes [64]. It must be noted that the majority of studies have been conducted on healthy adults and there is inconsistency in terms of supplementation protocols, duration and also a wide variety of exercise protocols have been utilised. Antioxidants reduce OS, play a key role in immunity and may improve recovery following exercise [58,59]. Further research is necessary to investigate the recovery promoting doses of antioxidants within athletic populations [38,65]. The potential sleep promoting benefits of antioxidant consumption/supplementation should be investigated also.

#### 2.6.1. Tart Cherries

Tart cherries contain high concentrations of melatonin and a range of phenolic compounds that have both antioxidant and anti-inflammatory properties [66,67]. A recent study was conducted to investigate the effect of tart cherry juice (2 × servings of 30 mL concentrate) on sleep enhancement, sleep duration and sleep quality [50]. This was the first investigation to demonstrate that tart cherry juice supplementation increased circulating melatonin levels and improved sleep time and quality in healthy adults. In the intervention group tart cherry juice supplementation resulted in significantly elevated total melatonin content, increased time in bed (+24 min), increased total sleep duration (+34 min) improved sleep efficiency total (82.3%) and a significant reduction in daytime napping (–22%) (*p* < 0.05) [50]. It must be noted that elevated melatonin concentrations may not be only mechanism at work as sleep regulation is also influenced by proinflammatory cytokines [3]. Tart cherries also contain numerous compounds that have antioxidant and anti-inflammatory properties. A similar study demonstrated that tart cherry juice consumption resulted in significantly reduced insomnia severity index scores (13.2 ± 2.8 versus control 14.9 ± 3.6; *p* < 0.05) and wake after sleep onset time (62.1 ± 37.4 min versus control, 79.1 ± 38.6 mins; *p* < 0.01), in older females with insomnia (*n* = 7) compared to a placebo [68]. 

Indeed, there is evidence that tart cherry juice supplementation post exercise may aid recovery from running a marathon [67]. The intervention group demonstrated a more rapid return of baseline isometric knee extension strength (pre-race 432 ± 114 vs. 48h 435 ± 109), 48 h post-marathon which was not demonstrated in the control group (pre-race 384 ± 112 vs. 48 h 349 ± 96) [67], indicating that consumption of tart cherry juice may blunt the secondary muscle damage response (localised inflammation). Post-race levels of inflammation were significantly reduced in the intervention group (IL-6 41.8 pg/mL) compared to the control group (IL-6 82.1 pg/mL) [67]. Similarly, post-race elevations in CRP and uric acid were significantly reduced in the intervention group (*p* < 0.001) [67]. Total antioxidant capacity was increased in both groups post-race (intervention 124% of baseline and control 112% of baseline, *p* < 0.01) and remained elevated at 24 h in the intervention group (114% of baseline) but not the control group [67]. During recovery athletes can suffer from delayed onset muscle soreness (DOMS) [66], which can reduce sleep quantity and quality. A recent study has demonstrated tart cherry juice supplementation (30 mL, twice per day for seven days) reduced the post-exercise decline in functional performance following intermittent sprint activity (maximal voluntary isometric contractions, 20 m sprint, counter movement jump and 505 agility test), DOMS and inflammatory response (Il-6) [66]. With regards the reduction in both DOMS and the post-exercise inflammatory response, in practice, the researchers suggested that this might be beneficial during periods of high-volume training (e.g., pre-season) or where athletes are required to produce multiple performances in a short space of time (e.g., double training sessions), when recovery periods are short [66]. The range of phenolic compounds in cherries which have anti-inflammatory and antioxidant properties may enhance post exercise recovery as well as sleep [50]. It has been proposed that melatonin may be synthesised in mitochondria, making melatonin and its metabolites available to protect the muscle against oxidative stress. Melatonin also increases the protective effects of glutathione, vitamin C and trolox, through regeneration by electron transfer processes [69].

#### 2.6.2. Kiwifruit

Kiwifruit are nutritionally dense containing a range of nutrients that can benefit sleep, health and recovery including serotonin, vitamin C, vitamin E, vitamin K, folate, anthocyanidins, carotenoids, beta-carotene, lutein, potassium, copper and fibre [70]. Interest in the antioxidant capacity, enzyme, polyphenolic and phytochemical content of kiwifruit has increased steadily over the last decade [70,71,72]. It has been suggested that the various bioactive components in kiwifruit may act synergistically affecting various physiological and metabolic processes (e.g., inhibition of oxidative and inflammatory responses, improved gastrointestinal tract health and bowel function) [73]. Contemporary research has focused on the health benefits of kiwifruit particularly in relation to antioxidant capacity, digestion, iron nutrition, metabolic health and immune function [73].

Regular consumption of kiwifruit has been found to significantly (*p* ≤ 0.05) increase plasma vitamin C [74] vitamin E [71] and lutein/zeaxanthin concentrations [71,74]. A study involving volunteers (*n* = 25) with a self-reported sleep disturbance demonstrated consumption of two kiwifruit one hour before bedtime for four weeks significantly improved actigraphy-measured total sleep duration (+16.9%, baseline 354.5 ± 17.1 min; post-intervention 395.3 ± 17.4 min) and sleep efficiency (+2.4%, baseline 93.9 ± 1.03 min; post-intervention 95.9 ± 0.67 min) (*p* ≤ 0.005) [70]. Self-report measures also improved significantly, wake after sleep onset reduced (time awake during sleep period) (–28.9%), sleep onset latency reduced (–35.4%) while sleep efficiency increased (5.4%) (*p* ≤ 0.002) [70]. Sleep quality was significantly improved following the four-week kiwifruit intervention however, the lack of a control group must be noted and there was a high level of subject drop out (*n* = 5). The findings may be prone to bias as subjects were recruited based on interest in participation in a dietary intervention study relating to sleep.

Serotonin is the end product of L-tryptophan metabolism and is related to REM [70]. The serotonin content in kiwifruit may contribute to improved sleep while the rich antioxidant content may supress free radical expression and inflammatory cytokines. Folate deficiency has been linked to insomnia (difficulty initiating or maintaining sleep, extended periods of wakefulness and/or insufficient sleep) and restless leg syndrome (repeated movement of or undesirable sensations in legs leading to sleep disruption) [70], the high folate content in kiwifruit may improve folate status and consequently improve sleep [70]. Although folates are widely consumed in the diet, they are destroyed by cooking or processing, however, kiwifruit are typically consumed in their raw form. Further research is warranted within athletic populations to investigate the potential sleep promoting properties of kiwifruit and the effect of kiwifruit consumption on both sleep quality, sleep quantity and recovery. 

### 2.7. B Vitamins and Magnesium

Vitamin B_12_ contributes to melatonin secretion, pyridoxine (vitamin B_6_) is involved in the synthesis of serotonin from tryptophan and niacin (vitamin B_3_) may elicit a tryptophan sparing effect [40]. Niacin can be synthesised endogenously from tryptophan via the Kynurenine Pathway, therefore, consuming a sufficient amount of niacin is necessary to inhibit 2,3-dioxygenase activity eliciting a tryptophan sparing effect, increasing its availability for synthesis serotonin and melatonin [40]. Folate (vitamin B_9_) and pyridoxine are involved in the conversion of tryptophan into serotonin [45]. The reduced form of folate (5-methyltetratrahydrofolate) increases tetrahydrobiopterin is a co-enzyme of tryptophan-5-hydroxylase which converts tryptophan into 5-hydroxytryptamine (5-HT) [45]. Pyridoxine’s role in the conversion of tryptophan into serotonin is related to the amino acid decarboxylase which speeds up the conversion rate of 5-HT to serotonin [45]. Mixed effects have been observed, with different doses of cobalamin (vitamin B_12_) on sleep-wake rhythms and delayed sleep phase syndrome (a significant delay in circadian rhythm), while no effect was observed for sleep duration [41].

Magnesium is also believed to enhance melatonin secretion promoting sleep onset and act as a GABA agonist, the main inhibitory neurotransmitter that acts on the CNS [40]. Magnesium is important for the production of the enzyme N-acetyltransferase which converts 5-HT into N-acetyl-5-hydroxytryptamine, which can then be converted to melatonin [44]. A placebo controlled double blind study on older adults (*n* = 43), demonstrated a food based supplement containing 5 mg melatonin, 225 mg magnesium and 11.25 mg zinc, significantly (*p* < 0.001) improved subjective sleep quality scores in the intervention group but not the controls (difference between the groups 6.8; 95% CI 5.4–8.3) and total sleep duration (182.18 min; 95% CI 160.02–204.34) assessed via actigraphy [75]. The effects were attributed to the synergy between magnesium, zinc and melatonin [75]. It must be noted that supplementing these nutrients will most likely only have an effect in cases of deficiency or insufficiency. 

## 3. Conclusions

Nutrients such as antioxidants, tryptophan rich protein, carbohydrate, melatonin, micronutrients and fruit can affect sleep [37,39,40]. Sleep can be promoted either by inhibiting wake-promoting mechanisms or by increasing sleep promoting factors through nutritional interventions [40]. Based on this review of the existing scientific literature, there appears to be considerable scope for further investigation of nutrition interventions designed to enhance sleep quality and quantity or promote general health, sleep health, training adaptations and/or recovery in both general and athletic populations. 

## Figures and Tables

**Table 1:** Characteristics of NREM Sleep.

Stage	Characteristics
1	Sleep is easily discontinued (e.g., noise, a light touch, etc.) Sleep is easily interrupted Key role in the initial wake to sleep transition Transitional stage throughout the sleep cycle
2	More intense stimuli required to produce arousal (e.g., bright light or loud noise) Indicated by K-complexes or sleep spindles in the EEG High voltage slow wave EEG activity will become apparent
3	High voltage (75 μV) slow wave (two cycles per second [cps]) activity that is ≥ 20% but < 50% of EEG activity
4	High voltage slow wave activity is ≥ 50% of EEG activity.

(Adapted from: [4]).

## References

[1] Halson S.L. (2014). Sleep in elite athletes and nutritional interventions to enhance sleep. Sports Med..

[2] Carskadon M.A., Dement W.C., Kryger M.H., Roth R., Dement W.C. (2011). Monitoring and Staging Human Sleep. Principles and Practice of Sleep Medicine.

[3] Irwin M.R., Opp M. (2017). Sleep health: Reciprocal regulation of sleep and innate immunity. Neuropsychopharmacology.

[4] Kryger M.H., Roth T., Dement W.C. (2011). Principles and Practice of Sleep Medicine.

[5] Berry R.B., Brooks R., Gamaldo C.E., Harding S.M., Lloyd R.M., Marcus C.L., Vaughn B.V. (2017). The AASM Manual for the Scoring of Sleep and Associated Events: Rules, Terminology and Technical Specifications: Version 2.3.

[6] Buysse D.J. (2014). Sleep health: Can we define it? Does it matter?. Sleep.

[7] Saper C.B., Scammell T.E., Lu J. (2005). Hypothalamic regulation of sleep and circadian rhythms. Nature.

[8] Saper C.B., Chou T.C., Scammell T.E. (2001). The sleep switch: Hypothalamic control of sleep and wakefulness. Trends Neurosci..

[9] McGinty D., Szymusiak R., Kryger M.H., Roth R., Dement W.C. (2005). Sleep-promoting mechanisms in mammals. Principles and Practice of Sleep Medicine.

[10] Czeisler C.A., Duffy J.F., Shanahan T.L., Brown E.N., Mitchell J.F., Rimmer D.W., Ronda J.M., Silva E.J., Allan J.S., Emens J.S. (1999). Stability, precision, and near-24-hour period of the human circadian pacemaker. Science.

[11] Rosenthal L., Day R., Gerhardstein R., Meixner R., Roth T., Guido P., Fortier J. (2001). Sleepiness/alertness among healthy evening and morning type individuals. Sleep Med..

[12] Vitale J.A., Weydahl A. (2017). Chronotype, Physical Activity, and Sport Performance: A Systematic Review. Sports Med..

[13] Borbély AA. (1982). A two-process model of sleep regulation. Hum. Neurobiol..

[14] Borbély A.A., Daan S., Wirz-Justice A., Deboer T. (2016). The two-process model of sleep regulation: A reappraisal. J. Sleep Res..

[15] Venter R.E. (2012). Role of sleep in performance and recovery of athletes: A review article. South Afr. J. Res. Sport Phys. Educ. Recreat..

[16] Rawashdeh O., Hudson R.L., Stepien I., Dubocovich M.L. (2011). Circadian periods of sensitivity for ramelteon on the onset of running-wheel activity and the peak of suprachiasmatic nucleus neuronal firing rhythms in C3H/HeN mice. Chronobiol. Int..

[17] Åkerstedt T., Folkard S. (1997). The three-process model of alertness and its extension to performance, sleep latency, and sleep length. Chronobiol. Int..

[18] Irwin M.R., Olmstead R., Carroll J.E. (2016). Sleep disturbance, sleep duration, and inflammation: A systematic review and meta-analysis of cohort studies and experimental sleep deprivation. Biol. Psychiatry.

[19] Frank M.G. (2006). The mystery of sleep function: Current perspectives and future directions. Rev. Neurosci..

[20] Irwin M.R. (2015). Why sleep is important for health: A psychoneuroimmunology perspective. Ann. Rev. Psychol..

[21] Dimitrov S., Besedovsky L., Born J., Lange T. (2015). Differential acute effects of sleep on spontaneous and stimulated production of tumour necrosis factor in men. Brain Behav. Immun..

[22] Vgontzas A.N., Fernandez-Mendoza J., Liao D., Bixler E.O. (2013). Insomnia with objective short sleep duration: The most biologically severe phenotype of the disorder. Sleep Med. Rev..

[23] Barzilay J.I., Forsberg C., Heckbert S.R., Cushman M., Newman A.B. (2006). The association of markers of inflammation with weight change in older adults: The Cardiovascular Health Study. Int. J. Obes..

[24] Irwin M.R., Cole S.W. (2011). Reciprocal regulation of the neural and innate immune systems. Nat. Rev. Immunol..

[25] Cappuccio F.P., D’Elia L., Strazzullo P., Miller M.A. (2010). Sleep duration and all-cause mortality: A systematic review and meta-analysis of prospective studies. Sleep.

[26] Golem D.L., Martin-Biggers J.T., Koenings M.M., Davis K.F., Byrd-Bredbenner C. (2014). An integrative review of sleep for nutrition professionals. Adv. Nutr..

[27] Johnston JD. (2014). Physiological links between circadian rhythms, metabolism and nutrition. Exp. Physiol..

[28] Samuels C., James L., Lawson D., Meeuwisse W. (2016). The Athlete Sleep Screening Questionnaire: A new tool for assessing and managing sleep in elite athletes. Br. J. Sports Med..

[29] Hausswirth C., Louis J., Aubry A., Bonnet G., Duffield R., Le Meur Y. (2014). Evidence of disturbed sleep and increased illness in overreached endurance athletes. Med. Sci. Sports Exerc..

[30] Hynynen E.S.A., Uusitalo A., Konttinen N., Rusko H. (2006). Heart rate variability during night sleep and after awakening in overtrained athletes. Med. Sci. Sports Exerc..

[31] Dattilo M., Antunes H.K.M., Medeiros A., Neto M.M., Souza H.S.D., Tufik S., De Mello M.T. (2011). Sleep and muscle recovery: Endocrinological and molecular basis for a new and promising hypothesis. Med. Hypotheses.

[32] Tuomilehto H., Vuorinen V.P., Penttilä E., Kivimäki M., Vuorenmaa M., Venojärvi M., Airaksinen O., Pihlajamäki J. (2017). Sleep of professional athletes: Underexploited potential to improve health and performance. J. Sports Sci..

[33] Erlacher D., Ehrlenspiel F., Adegbesan O.A., El-Din H.G. (2011). Sleep habits in German athletes before important competitions or games. J. Sports Sci..

[34] Milewski M.D., Skaggs D.L., Bishop G.A., Pace J.L., Ibrahim D.A., Wren T.A., Barzdukas A. (2014). Chronic lack of sleep is associated with increased sports injuries in adolescent athletes. J. Paediatr. Orthop..

[35] Frohm A., Kottorp A., Fridén C., Heijne A., Von Rosen P. (2016). Too little sleep and an unhealthy diet could increase the risk of sustaining a new injury in adolescent elite athletes. Scan. J. Med. Sci. Sports.

[36] Thomas D.T., Erdman K.A., Burke L.M. (2016). American College of Sports Medicine Joint Position Statement. Nutrition and Athletic Performance. Med. Sci. Sports Exerc..

[37] Jeukendrup A.E. (2017). Periodized Nutrition for Athletes. Sports Med..

[38] Close G.L., Hamilton D.L., Philp A., Burke L.M., Morton J.P. (2016). New strategies in sport nutrition to increase exercise performance. Free Radic. Biol. Med..

[39] Tahara Y., Shibata S. (2014). Chrono-biology, Chrono-pharmacology and Chrono-nutrition. J. Pharmacol. Sci..

[40] Peukhuri K., Sihvola N., Korpela R. (2012). Diet promotes sleep duration and quality. Nutr. Res..

[41] Roehrs T., Roth T. (2001). Sleep, sleepiness, and alcohol use. Alcohol. Res. Health.

[42] Hirshkowitz M., Whiton K., Albert S.M., Alessi C., Bruni O., DonCarlos L., Hazen N., Herman J., Katz E.S., Kheirandish-Gozal L. (2015). National Sleep Foundation’s sleep time duration recommendations: Methodology and results summary. Sleep Health.

[43] Silber B.Y., Schmitt J.A.J. (2010). Effects of tryptophan loading on human cognition, mood, and sleep. Neurosci. Biobehav. Rev..

[44] Afaghi A., O’connor H., Chow C.M. (2007). High-glycemic-index carbohydrate meals shorten sleep onset. Am. J. Clin. Nutr..

[45] Ordóñez F.M., Oliver A.J.S., Bastos P.C., Guillén L.S., Domínguez R. (2017). Sleep improvement in athletes: Use of nutritional supplements. Am. J. Sports Med..

[46] Grandner M.A., Jackson N., Gerstner J.R., Knutson K.L. (2014). Sleep symptoms associated with intake of specific dietary nutrients. J. Sleep Res..

[47] Porter JM., Horne JA. (1981). Bed-time food supplements and sleep: Effects of different carbohydrate levels. Electroencephalogr. Clin. Neurophysiol..

[48] Ramis M., Esteban S., Miralles A., Tan D.-X., Reiter R. (2015). Protective effects of melatonin and mitochondria-targeted antioxidants against oxidative stress: A review. Curr. Med. Chem..

[49] Bonnefont-Rousselot D., Collin F. (2010). Melatonin: Action as antioxidant and potential applications in human disease and aging. Toxicology.

[50] Howatson G., Bell P.G., Tallent J., Middleton B., McHugh M.P., Ellis J. (2012). Effect of tart cherry juice (Prunus Cerasus) on melatonin levels and enhanced sleep quality. Eur. J. Nutr..

[51] Milagres M.P., Minim V.P., Minim L.A., Simiqueli A.A., Moraes L.E., Martino H.S. (2014). Night milking adds value to cow’s milk. J. Sci. Food Agric..

[52] Pires M.L.N., Benedito-Silva A.A., Pinto L. (2001). Acute effects of low doses of melatonin on the sleep of young healthy subjects. J. Pineal Res..

[53] Hudson C., Hudson S.P., Hecht T., MacKenzie J. (2005). Protein source tryptophan versus pharmaceutical grade tryptophan as an efficacious treatment for chronic insomnia. Nutr. Neurosci..

[54] Markus C.R., Olivier B., Panhuysen G.E., Van der Gugten J., Alles M.S., Tuiten A., Westenberg H.G., Fekkes D., Koppeschaar H.F., de Haan E.E. (2000). The bovine protein α-lactalbumin increases the plasma ratio of tryptophan to the other large neutral amino acids, and in vulnerable subjects raises brain serotonin activity, reduces cortisol concentration, and improves mood under stress. Am. J. Clin. Nutr..

[55] Markus C.R., Jonkman L.M., Lammers J.H., Deutz N.E., Messer M.H., Rigtering N. (2005). Evening intake of α-lactalbumin increases plasma tryptophan availability and improves morning alertness and brain measures of attention. Am. J. Clin. Nutr..

[56] Arnulf I., Quintin P., Alvarez J.C., Vigil L., Touitou Y., Lèbre A.S., Bellenger A., Varoquaux O., Derenne J.P., Allilaire J.F. (2002). Mid-morning tryptophan depletion delays REM sleep onset in healthy subjects. Neuropsychopharmacology.

[57] Bhatti T., Gillin J.C., Seifritz E., Moore P., Clark C., Golshan S., Stahl S., Rapaport M., Kelsoe J. (1998). Effects of a tryptophan-free amino acid drink challenge on normal human sleep electroencephalogram and mood. Biol. Psychiatry.

[58] Halliwell B., Gutteridge J.M. (2015). Free Radicals in Biology and Medicine.

[59] Nieman D.C., Mitmesser S.H. (2017). Potential Impact of Nutrition on Immune System Recovery from Heavy Exertion: A Metabolomics Perspective. Nutrients.

[60] Nieman D.C., Henson D.A., Mcanulty S.R., Mcanulty L.S., Morrow J.D., Ahmed A., Heward C.B. (2004). Vitamin E and immunity after the Kona triathlon world championship. Med. Sci. Sports Exerc..

[61] Finaud J., Lac G., Filaire E. (2006). Oxidative stress. Sports Med..

[62] Cobley J.N., Margeritelis N.V., Morton J.P., Close G.L., Nikolaidis M.G., Malone J.K. (2015). The basic chemistry of exercise induced DNA oxidation: Oxidative damage, redox signalling, and their interplay. Front. Physiol..

[63] Cobley J.N., McHardy H., Morton J.P., Nikolaidis M.G., Close G.L. (2015). Influence of Vitamin C and Vitamin E on redox signalling: Impications for exercise adaptations. Free Radic. Biol. Med..

[64] Mankowski R.T., Anton S.D., Buford T.W., Leeuwenburgh C. (2015). Dietary antioxidants as modifiers of physiologic adaptations to exercise. Med. Sci. Sports Exerc..

[65] Pritchett K., Moore A. (2018). Food with Benefits: Gain the Competitive Edge With a “Food-First” Approach. ACSM’s Health Fit. J..

[66] Bell P., Stevenson E., Davison G., Howatson G. (2016). The effects of Montmorency tart cherry concentrate supplementation on recovery following prolonged, intermittent exercise. Nutrients.

[67] Howatson G., McHugh M.P., Hill J.A., Brouner J., Jewell A.P., Van Someren K.A., Shave R.E., Howatson S.A. (2010). Influence of tart cherry juice on indices of recovery following marathon running. Scan. J. Med. Sci. Sports.

[68] Pigeon W.R., Carr M., Gorman C., Perlis M.L. (2010). Effects of a tart cherry juice beverage on the sleep of older adults with insomnia: A pilot study. J. Med. Food.

[69] Galano A., Castañeda-Arriaga R., Pérez-González A., Tan D.X., Reiter R. (2016). Phenolic Melatonin-Related Compounds: Their Role as Chemical Protectors against Oxidative Stress. Molecules.

[70] Lin H.H., Tsai P.S., Fang S.C., Liu J.F. (2011). Effect of kiwifruit consumption on sleep quality in adults with sleep problems. Asia Pac. J. Clin. Nutr..

[71] Hunter D.C., Skinner M.A., Wolber F.M., Booth C.L., Loh J.M., Wohlers M., Stevenson L.M., Kruger M.C. (2012). Consumption of gold kiwifruit reduces severity and duration of selected upper respiratory tract infection symptoms and increases plasma vitamin C concentration in healthy older adults. Br. J. Nutr..

[72] Thorpy M.J. (2012). Classification of sleep disorders. Neurotherapeutics.

[73] Singletary K. (2012). Kiwifruit: Overview of potential health benefits. Nutr. Today.

[74] Beck K., Conlon C.A., Kruger R., Coad J., Stonehouse W. (2011). Gold kiwifruit consumed with an iron-fortified breakfast cereal meal improves iron status in women with low iron stores: A 16-week randomised controlled trial. Br. J. Nutr..

[75] Rondanelli M., Opizzi A., Monteferrario F., Antoniello N., Manni R., Klersy C. (2011). The effect of melatonin, magnesium, and zinc on primary insomnia in long-term care facility residents in Italy: A double-blind, placebo-controlled clinical trial. J. Am. Geriatr. Soc..

